# Bioactive Compounds in Wild Nettle (*Urtica dioica* L.) Leaves and Stalks: Polyphenols and Pigments upon Seasonal and Habitat Variations

**DOI:** 10.3390/foods10010190

**Published:** 2021-01-18

**Authors:** Maja Repajić, Ena Cegledi, Zoran Zorić, Sandra Pedisić, Ivona Elez Garofulić, Sanja Radman, Igor Palčić, Verica Dragović-Uzelac

**Affiliations:** 1 Faculty of Food Technology and Biotechnology, University of Zagreb Pierottijeva 6, 10000 Zagreb, Croatia; maja.repajic@pbf.unizg.hr (M.R.); ecegledi@pbf.hr (E.C.); zzoric@pbf.hr (Z.Z.); sandra.pedisic@pbf.unizg.hr (S.P.); vdragov@pbf.hr (V.D.-U.); 2 Faculty of Agriculture, University of Zagreb, Svetošimunska cesta 25, 10000 Zagreb, Croatia; sradman@agr.hr; 3 Institute of Agriculture and Tourism, Karla Huguesa 8, 52440 Poreč, Croatia; palcic@iptpo.hr

**Keywords:** nettle leaves and stalks, phenological stage, location, accelerated solvent extraction, UPLC-MS/MS, polyphenols, chlorophylls, carotenoids, antioxidant capacity, ORAC

## Abstract

This study evaluated the presence of bioactives in wild nettle leaves and stalks during the phenological stage and in the context of natural habitat diversity. Thus, wild nettle samples collected before flowering, during flowering and after flowering from 14 habitats situated in three different regions (continental, mountain and seaside) were analyzed for low molecular weight polyphenols, carotenoids and chlorophylls using UPLC-MS/MS and HPLC analysis, while the ORAC method was performed for the antioxidant capacity measurement. Statistical analysis showed that, when compared to the stalks, nettle leaves contained significantly higher amounts of analyzed compounds which accumulated in the highest yields before flowering (polyphenols) and at the flowering stage (pigments). Moreover, nettle habitat variations greatly influenced the amounts of analyzed bioactives, where samples from the continental area contained higher levels of polyphenols, while seaside region samples were more abundant with pigments. The levels of ORAC followed the same pattern, being higher in leaves samples collected before and during flowering from the continental habitats. Hence, in order to provide the product’s maximum value for consumers’ benefit, a multidisciplinary approach is important for the selection of a plant part as well as its phenological stage with the highest accumulation of bioactive compounds.

## 1. Introduction

Nettle (*Urtica dioica* L.) is a perennial wild plant of the Urticaceae family, genus Urtica, which is widespread in Europe, Asia, America and part of Africa, and has been adapted to different climatic conditions [1,2]. Nettle has long been used in the food, cosmetic and pharmaceutical industries due to its nutritional and health potential, as all parts of nettle (leaves, stalks and roots) show a rich composition of bioactive compounds with high antioxidant capacity [2,3] Previous studies have shown that nettle leaves and stalks are a rich source of vitamins A, B and C, minerals (iron, potassium, calcium, magnesium), polyphenols such as phenolic acids and flavonoids as well as pigments, especially chlorophyll and carotenoids [4,5,6,7,8,9,10,11]. In accordance with the above, aerial parts of nettle have anti-inflammatory and therapeutic effects; these nettle parts are used in the treatment of arthritis, anemia, allergies, joint pain and urinary tract infections, have a diuretic effect and are used to strengthen hair [3,12]. Besides aerial parts, nettle root also presents a rich source of various compounds such as protein lectin, sterols, polysaccharides, lignans and phenols [5,7,13,14] and is mostly used in the treatment of benign prostatic hyperplasia [15]. Apart from medicinal use, other applications of nettle include food preparation, where it is consumed in the form of tea, soup, stew or salad [3], or for commercial extraction of chlorophyll, which is used as a green coloring agent (E140) [16].

For medicinal purpose and medicinal preparations, nettle is mostly often used in the form of liquid or dry extract; thus, it is important to apply extraction method that will give a highly stabile extract with the greatest possible content of bioactive ingredients. Therefore, new extraction methods are increasingly being used and one of them is accelerated solvent extraction (ASE). In addition to being an efficient method, it uses less solvent, shortens the extraction time and more effectively isolates the target components [17].

Aside from extraction method, extract quality and richness in bioactives also depends on used plant material, either wild or cultivated, where its chemical composition and consequently antioxidant capacity are influenced by environmental, genotypic and phenotypic factors.

Different parts of plant may contain different amounts of particular compounds, e.g., nettle leaves accumulate higher amounts of polyphenols and chlorophylls in comparison with stalks [6,7,18]. In general, leaves are the richest part of a nettle in bioactive compounds, therefore they are mostly used in processing. However, changes in chemical composition and compounds’ distribution occur with plant’s maturity, where bioactive compounds are present in different proportions during different phenological stages. For example, the content of polyphenols decreases with growth and maturity of the plant [19]. Bioactive compounds are produced in response to different forms of (a)biotic stresses, as well as to fulfil important physiological tasks (attracting pollinators, establishing symbiosis, providing structural components to lignified cell walls of vascular tissues, etc.) [20]. These processes are often connected to specific phenological stages. Hence, harvest time depends on the type of final product. Although opinion on nettle optimal harvest time differs among various authors [3], Moore (1993) [21] stated that for juices and other fresh preparations, nettle leaves are best picked in spring or early summer (before flowering), and according to Upton (2013) [3] for dried preparations, it is best to harvest from mid-spring to late summer. If nettle is used for food purposes, the recommended harvest should be at the pre-flowering and flowering phases, certainly before the appearance of the seeds when it contains the least bioactive ingredients [3].

Nevertheless, nettle herb is mostly wild-harvested [3]. Concerning the natural habitat and climate, nettle is a quite adaptable plant. It grows in areas characterized by mild to temperate climates and prefers open or partly shady habitats with plenty of moisture such as forests, by rivers or streams and on roadsides [2]. Still, accumulation of polyphenols and pigments varies upon climate and habitat diversity. Plants grown in cold climates often show greater antioxidant properties, as a result of oxidative stress defense [22], while pigments synthesis is enhanced due to exposure to higher temperatures and more sunlight [23,24].

Although mentioned scientific literature provides data regarding nettle chemical composition, to our best knowledge there are no comprehensive studies on polyphenols and pigments constituents and their accumulation in wild nettle leaves and stalks during different vegetation periods of growing across diverse regions. These cognitions could be beneficial input data in a production of liquid and dry extracts. Therefore, the current study aimed to examine the presence and profile of low molecular weight polyphenols, carotenoids and chlorophylls as well as to determine antioxidant capacity in wild nettle leaves and stalks collected during three phenological stages (before flowering, during flowering and after flowering) from 14 different natural habitats situated in three regions in Croatia.

## 2. Materials and Methods

### 2.1. Chemicals

HPLC grade acetonitrile was procured from J.T. Baker Chemicals (Deventer, Netherlands). Purified water was obtained by a Milli-Q water purification system (Millipore, Bedford, MA, USA). Ethanol (96%) was purchased from Gram–mol d.o.o. (Zagreb, Croatia) and formic acid (98–100%) from T.T.T. d.o.o. (Sveta Nedjelja, Croatia). Commercial standards of quercetin-3-glucoside, kaempferol-3-rutinoside, myricetin, caffeic acid, gallic acid, ferulic acid, sinapic acid, quinic acid, chlorogenic acid, *p*-coumaric acid, esculetin, scopoletin, α-carotene, β-carotene, chlorophyll *a* and chlorophyll *b* were purchased from Sigma–Aldrich (St. Louis, MO, USA). Epicatechin, catechin, epigallocatechin gallate, epicatechin gallate, apigenin, luteolin and naringenin were obtained from Extrasynthese (Genay, France), while quercetin-3-rutinoside was procured from Acros Organics (Thermo Fisher Scientific, Geel, Belgium). Apigenin was dissolved in ethanol with 0.5% (*v/v*) dimethyl sulfoxide, standards of carotenoids and chlorophylls in *n*-hexane. Other standards were prepared as a stock solution in methanol, and working standard solutions were prepared by diluting the stock solutions to yield five concentrations.

### 2.2. Plant Material

Samples of wild nettle (*Urtica dioica* L.) were collected at three phenological stages [(I) before flowering, (II) during flowering and (III) after flowering] during 2019 from different habitats in Croatia belonging to three regions (continental, mountain and seaside) (Table 1). Plant material was identified by using usual keys and iconographies with support of Department of Vegetable Crops, Faculty of Agriculture, University of Zagreb (Croatia). Immediately after harvesting, leaves were separated from stalks and samples were stored at −18 °C, freeze-dried (Alpha 1-4 LSCPlus, Martin Christ Gefriertrocknungsanlagen GmbH, Osterode am Harz, Germany) and afterwards grinded into fine powder using a commercial grinder (GT11, Tefal, Rumilly, France). Obtained powders were immediately analyzed for total solids by drying at 103 ± 2 °C to constant mass [25] and further used for the extraction. Content of dry matter in samples was >95%.

### 2.3. Extraction Conditions

Extraction of polyphenols and pigments from dry nettle leaves and stalks was carried out by ASE. Extraction conditions and procedure were adopted from the study of Repajić et al. (2020) [11]: extraction was performed in Dionex™ ASE™ 350 Accelerated Solvent Extractor (Thermo Fisher Scientific Inc., Sunnyvale, CA, USA) using ethanol (96%) as the extraction solvent. Extraction was accomplished in 34 mL stainless steel cells fitted with 2 cellulose filters (Dionex™ 350/150 Extraction Cell Filters, Thermo Fisher Scientific Inc., Sunnyvale, CA, USA), within which 1 g of sample was mixed with 2 g of diatomaceous earth, placed in cell and filled up with diatomaceous earth to the full cell volume. Extraction parameters differed for leaves and stalks: leaves were extracted under 110 °C with 10 min of static extraction time and 4 cycles, while stalk extracts were obtained at 80 °C, 5 min of static extraction time and 4 cycles (parameters previously optimized). Other extraction parameters remained fixed for the extraction of both plant parts, namely pressure 10.34 MPa, 30 s of purge with nitrogen and 50% of flushing. Obtained extracts were collected in 250 mL glass vessel with Teflon septa, transferred into 50 mL volume flask and made up to volume with the extraction solvent. All extracts were filtered through a 0.45 μm membrane filter (Macherey-Nagel GmbH, Düren, Germany) prior to further analysis. All extracts have been prepared in a duplicate (n = 2).

### 2.4. UPLC-MS/MS Conditions

Identification and quantification of phenolics were performed on UPLC–MS/MS in both ionization modes on a 6430 QQQ mass spectrometer Agilent Technologies (Agilent, Santa Clara, CA, USA). Analytes were ionized using ESI ion source with nitrogen as desolvation and collision gas (temperature 300 °C, flow 11 L h^−1^), capillary voltage, +4 −3.5 kV^−1^ and the pressure of nebulizer was set at 40 psi. The mass spectrometer was linked to UPLC system (Agilent series 1290 RRLC instrument) consisted of binary pump, autosampler and a column compartment thermostat. Reversed phase separation was performed on a Zorbax Eclipse Plus C18 column 100 × 2.1 mm with 1.8 µm particle size (Agilent). Column temperature was set at 35 °C and the injection volume was 2.5 µL. The solvent compositions and the gradient conditions used were as described previously by Elez Garofulić et al. (2018) [26]. For instrument control and data processing, Agilent MassHunter Workstation Software (ver. B.04.01) was used. Quantitative determination was carried out using the calibration curves of the standards, where protocatechuic acid, gentisic acid, syringic acid and *p*-hydroxybenzoic acid were calculated as gallic acid equivalents and cinnamic acid according to *p*-coumaric acid. Isorhamnetin rutinoside, quercetin rhamnoside, quercetin, isorhamnetin, quercetin pentoside, quercetin acetylhexoside, quercetin acetylrutinoside and quercetin pentosylhexoside were calculated according to quercetin-3-glucoside, kaempferol hexoside, kaempferol pentoside, kaempferol rhamnoside, kaempferol pentosylhexoside and kaempferol according to kaempferol-3-rutinoside, apigenin hexoside and genistein according to apigenin, while umbelliferone was expressed as scopoletin equivalents. All analyses have been performed in a duplicate and concentrations of analyzed compounds are expressed as mg 100 g^−1^ of dry matter (dm) (N = 4).

### 2.5. HPLC-UV-VIS/PDA Conditions

The carotenoids and chlorophylls identification and quantification were performed using Agilent Infinity 1260 system equipped with Agilent 1260 photodiode array detector (PDA; Agilent, Santa Clara, CA, USA) with an automatic injector and Chemstation software (ver. C.01.03).

The separation of carotenoids and chlorophylls was performed using Develosil RP-Aqueus C 30 column (250 × 4.6 mm i.d. 3 µm, Phenomenex, Torrance, CA, USA). The solvent composition and the used gradient conditions were described previously by Castro–Puyana et al. (2017) [27]. The mixture of MeOH:MTBE:water (90:7:3, *v/v/v*) (A) and MeOH:MTBE (10:90, *v/v*) (B) formed the mobile phase. The injection volume was 10 µL and the flow rate was kept at 0.8 mL min^−1^. The chromatogram was monitored by scanning from 240 to 770 nm and the signal intensities detected at 450 nm and 660 nm were used for carotenoid and chlorophyll quantitation. Identification was carried out by comparing retention times and spectral data with those of the authentic standards (α- and β-carotene, chlorophyll *a* and *b*) or in case of unavailability of standards by comparing the absorption spectra reported in the literature [28,29]. Quantifications were made by the external standard calculation, using calibration curves of the standards α-carotene, β-carotene, chlorophyll *a* and chlorophyll *b*. The quantification of individual carotenoid compounds (neoxantine, violaxantine, lutein and its derivatives, derivative of zeaxantine and lycopene) was calculated as β-carotene equivalents and derivatives of chlorophylls as chlorophyll *a* and *b* equivalents using the equation based on the calibration curves, respectively. All determinations have been performed in a duplicate and results are expressed as mg 100 g^−1^ dm (N = 4).

### 2.6. ORAC Determination

The procedure was based on a previously reported method [30,31] with slight modifications. Briefly, a 96 wells black microplate was prepared containing 150 µL of fluorescein solution (70.30 nM) and 25 µL of blank (75 µM phosphate buffer, pH 7.4), Trolox standard (3.24–130.88 µM) or sample (appropriate diluted) were added. The plate was incubated for 30 min at 37 °C. After the first three cycles (representing the baseline signal), AAPH (240 mM) was injected into each well to initiate the peroxyl radical generation. Fluorescence intensity (excitation at 485 nm and emission at 528 nm) was monitored every 90 sec over a total measurement period of 120 min using an automated plate reader (BMG LABTECH, Offenburg, Germany) and data were analyzed by MARS 2.0 software. The results were expressed as mmol Trolox equivalent (TE) 100 g^−1^ of dm. Determinations were carried out in duplicate (N = 4).

### 2.7. Statistical Analysis

Statistica ver. 10.0 software (Statsoft Inc., Tulsa, OK, USA) was applied for the statistical analysis. Full factorial randomized design was designated for the experimental part and descriptive statistic was used for the basic data evaluation. Continuous variables (polyphenols, pigments and antioxidant capacity) were analyzed by multifactorial analysis of variance (MANOVA) and marginal mean values were compared with Tukey’s HSD test. Relationships between determined compounds and antioxidant capacity were examined by calculated Pearson’s correlation coefficients, while possible grouping of the samples according to the examined sources of variations was tested using Principal Component Analysis (PCA). Significance level *p* ≤ 0.05 was assigned for all tests.

## 3. Results and Discussion

This study examined the influence of plant part (leaves and stalks), phenological stage (before flowering, during flowering and after flowering) and habitat (Table 1) on the concentrations of polyphenols and pigments in wild nettle grown in Croatia. A total of 84 nettle samples were analyzed, where target compounds (polyphenols and pigments) were extracted using ASE and their identification/quantification was assessed by UPLC-MS/MS (polyphenols) and HPLC-UV-VIS/PDA (pigments). Moreover, obtained extracts were characterized for their antioxidant capacity by the ORAC method.

### 3.1. Influence of Phenological Stage and Habitat on Polyphenols in Nettle Leaves and Stalks

Table 2 shows detailed polyphenolic profile and mass spectrometric data obtained by UPLC-MS/MS analysis of nettle leaves and stalks. A total of 41 polyphenolic compounds were identified, belonging to the classes of benzoic, cinnamic and other phenolic acids, flavonols, flavan-3-ols, flavones, isoflavones, flavanones and coumarins (Supplementary files 1 & 2). Among the benzoic acids, compound **35** was identified as gallic acid by comparison of its retention time and mass spectra data with those of an authentic standard. Other benzoic acids were tentatively identified according to their mass fragmentation patterns. Compounds **2** and **14** showed same fragmentation pattern with molecular ion at *m/z* 153 and fragment ion at *m/z* 109, corresponding to the loss of carbon dioxide moiety and implicating the structure of dihydroxybenzoic acids and were therefore according to their polarity tentatively identified as protocatehuic (3,4-dihydroxybenzoic acid) and gentisic acid (1,3-dihydroxybenzoic acid), respectively [32]. Compound **31** showed precursor ion at *m/z* 197 and fragmentation loss of −15 amu corresponding to the loss of methyl radical characteristic for methoxylated phenolic acids and was tentatively identified as syringic acid. Compound **34** showed precursor ion at *m/z* 137 and characteristic fragmentation pattern for deprotonated phenolic acid with loos of −44 amu due to decarboxylation [33] and was assigned as *p*-hydroxybenzoic acid. The composition of benzoic acids in nettle leaves and stalks is in accordance with previous reports [9,14]. Among the cinnamic acids, compounds **12**, **15**, **19**, **25** and **32** were identified using authentic standards as caffeic, chlorogenic, *p*-coumaric, ferulic and sinapic acid, respectively. Compound **21** was presented with precursor ion at *m/z* 147, and fragment ion at *m/z* 103 as a result of decarboxylation and was due to its mass spectra data assigned as cinnamic acid [32]. Compound **16** was identified as quinic acid comparing its spectral data and retention time with those of an authentic standard. The composition of cinnamic acids is in accordance with previous reports by Orčić et al. (2014) [14] and Francišković et al. (2017) [34] with the exception of cinnamic acid which was not detected in their research, but was reported previously in composition of nettle leaves by Zeković et al. (2017) [35]. The most numerous class of flavonoid polyphenols identified in nettle were flavonols and their glycosides. Compounds **4**, **8**, **17** and **18** were identified by the authentic standard comparison as kaempferol-3-rutinoside, myricetin, quercetin-3-glucoside and quercetin-3-rutinoside, respectively. Other compounds were tentatively identified according to their mass spectra and characteristic fragmentation patterns reported previously. Among the aglycones, compounds **10**, **24** and **41** were assigned as quercetin, isorhamnetin and kaempferol due to characteristic molecular ion at *m/z* 301, *m/z* 315 and *m/z* 285 [36]. The presence of this aglycones in nettle aerial parts was confirmed previously by Bucar et al. (2006) [37]. Flavonol glycosides lacking authentic standards were tentatively identified according to the characteristic loss of sugar moiety and formation of aglycon fragment ion. Therefore, because of fragment ion at *m/z* 317, compound **3** was assigned as isorhamnetin glycoside. Precursor ion at *m/z* 625 implicated glycosylation with rhamnose (+146 amu) and glucose (+162 amu), so it was assigned as isorhametin rutinoside.

Its presence in nettle leaves and stalks was reported previously by Pinelli et al. (2008) [6]. Compounds **5**, **28**, **30**, **33** and **36** were identified as quercetin glycosides due to MS/MS ion at *m/z* 303 and were assigned as quercetin rhamnoside, quercetin pentoside, quercetin acetylhexoside, quercetin acetylrutinoside and quercetin pentosylhexoside due to fragmentation losses corresponding to rhamnose (−146 amu), pentose (−132 amu), hexose with acetyl residue (−162 and −42 amu), rutinose with acetyl residue (−308 and −42 amu) and pentose with hexose moiety (−132 and −162 amu) [38]. Previous reports on quercetin glycosides composition in nettle mostly included quercetin glucoside [6,14,34] and quercetin rutinoside [8,14,34], while not reporting the presence of acylated glycosides and diglycosides identified in this study. The latter provides the valuable contribution to detailed insight into nettle polyphenolic profile. Because of the characteristic fragment ion at *m/z* 287 corresponding to the kaempferol aglycon, compounds **6**, **27**, **29** and **39** were assigned as kaempferol hexoside, pentoside, rhamnoside and pentosylhexoside, respectively, due to fragment losses of corresponding sugar moieties. Similar to the previous literature reports on quercetin glycosides, the ones on kaempferol glycosides mostly only include kaempferol rutinoside [6,8] or glucoside [14,34,39], while not reporting the presence of kaempferol pentoside, rhamnoside and pentosylhexoside which are therefore being confirmed here for the first time. All compounds belonging to the class of flavan-3-ols (**23**, **37**, **38** and **40**), namely epigallocatechingallate, epicatechin, catechin and epicatechingallate were identified and confirmed according to the authentic standard. Orčić et al. (2014) [14] identified catechin in nettle stalks, epicatechin was reported by Proestos et al. (2006) [40] in leaves, while there are no available reports on previous identification of epicatechingallate and epigallocatechingallate. Compounds **9** and **22** were assigned as luteolin and apigenin due to molecular ions at *m/z* 287 and *m/z* 271 and confirmed by comparison with standards, while compound **7** was tentatively identified as apigenin hexoside based on fragment ion at *m/z* 271 and fragmentation loss of -162 amu specific for hexose residue. Nencu et al. (2012) [41] reported the polyphenolic composition of nettle leaves including aglycones luteolin and apigenin, which is in accordance with our findings, while literature reports on flavone aglycones are scarce. Compound **20** showed precursor ion at *m/z* 269 and fragment ion at *m/z* 133, corresponding to the previously reported fragmentation mechanism of genistein anion [42], confirmed in nettle leaves extract by Zeković et al. (2017) [35]. Compounds **11**, **13** and **26** were identified by its corresponding authentic standards as naringenin, esculetin and scopoletin, while compound **1** was tentatively assigned as umbelliferone due to molecular ion at *m/z* 161 and fragment ion at *m/z* 133 formed after the loss of one carbon monoxide molecule [43]. The composition of flavanones and coumarines reported in our study is in accordance with previous literature data [14,34,35].

To examine the influence of phenological stage and habitat on the content of polyphenols in nettle leaves and stalks, identified polyphenols were arranged in corresponding classes, following which their individual concentrations accordingly summarized and subjected to statistical analysis, as shown in Table 3. Total polyphenols grand mean (GM) was 380.90 mg 100 g^−1^ dm, among which cinnamic acids were the most abundant group (GM 179.22 mg 100 g^−1^ dm), followed by flavonols (GM 134.60 mg 100 g^−1^ dm), flavones (GM 24.56 mg 100 g^−1^ dm), flavan-3-ols (GM 20.70 mg 100 g^−1^ dm) and benzoic acids (GM 10.20 mg 100 g^−1^ dm). Coumarins, isoflavones and other acids were present in lower concentrations: GM values 5.31, 3.09 and 2.88 mg 100 g^−1^ dm, respectively, while the least represented group of polyphenols were flavanones (GM 0.34 mg 100 g^−1^ dm). Moreover, obtained results are in accordance with the results of other authors [6,8,11,14], who reported quite similar phenolic profile in nettle extracts where cinnamic acids accounted for the most of presented total polyphenols.

As can be observed, the plant part, phenological stage and habitat had a significant influence (*p* < 0.01) on amounts of all polyphenols’ groups. When comparing amounts of polyphenols between nettle leaves and stalks, it can be seen that leaves accumulated significantly higher concentrations of all polyphenols’ groups (Table 3). Otles and Yalcin (2012) [7] also documented higher polyphenols content in wild nettle leaves extracts when compared to stalks extracts, as well as Pinelli et al. (2008) [6] who studied the content of polyphenols in cultivated and wild nettle and reported higher total polyphenols in leaves of both types of nettle (cultivated 7.364 mg g^−1^ fw, wild 2.58 mg g^−1^ fw) as opposed to nettle stalks (cultivated 3.670 mg g^−1^ fw, wild 0.750 mg g^−1^ fw).

Same authors documented the abundance of nettle stalks with fibers, consisting of several components of the lignin. However, in study of Orčić et al. (2014) [14], who examined nettle samples picked at three different locations, several identified polyphenols were recorded in higher levels in stalks, but the cinnamic acids presented in their study with chlorogenic acid were also more abundant in leaves.

Considering the phenological stage, it can be noticed that the 1st phenological stage (before flowering) resulted with higher concentrations of all polyphenols, except flavan-3-ols which were significantly higher during the 2nd phenological stage (flowering) (Table 3). Overall, total polyphenols decreased for almost 50% by the 3rd phenological stage. Similar to our results, in two studies of Nencu et al. (2012, 2013) [41,44], it was concluded that the optimal time for nettle leaves harvest was March, since the polyphenols content greatly decreased (over 80%) by June and September, respectively. Authors reported that the total polyphenols decrease is due to the decrease of non-tannin phenols (phenolcarboxylic acids and flavonoids), which are the most important compounds from nettle leaves. This was also confirmed by Roslon et al. (2003) [45] who reported a sudden drop of phenolcarboxylic acids in leaves harvested at the plant flowering stage. Furthermore, the results of Biesiada et al. (2009, 2010) [46,47] and Kőszegi et al. (2020) [19] also indicated that the beginning of the nettle vegetation period was optimal for harvesting, giving the highest yield of polyphenols, which then decreased by autumn for over 50%. Therefore, in order to obtain extracts with the highest polyphenols content, the optimal time to harvest the aerial parts of the nettle is spring (before the flowering of the plant). It can be assumed that the total polyphenols decrease starting at the flowering stage is a result of the physiological switch from the vegetative to the generative phase and the formation of flowers [48].

Habitats of wild nettle samples differed according to the climate conditions and could be grouped into three different regions: continental, mountain and seaside (Table 1). As presented in Table 3, habitats significantly (*p* < 0.01) differed regarding polyphenols content, with no uniform pattern regarding individual polyphenolic groups. Thus, Žakanje, belonging to the continental region, was characterized with the highest concentrations of total polyphenols (513.12 ± 1.03 mg 100 g^−1^ dm), benzoic (19.39 ± 0.10 mg 100 g^−1^ dm) and cinnamic acids (227.10 ± 0.70 mg 100 g^−1^ dm), flavan-3-ols (32.13 ± 0.11 mg 100 g^−1^ dm), flavones (42.44 ± 0.12 mg 100 g^−1^ dm) and isoflavones (5.29 ± 0.05 mg 100 g^−1^ dm). Contrarily, Ogulin, situated in mountain areas, was characterized with the highest amounts of other acids (4.62 ± 0.07 mg 100 g^−1^ dm), flavonols (182.65 ± 0.38 mg 100 g^−1^ dm), flavanones (0.56 ± 0.02 mg 100 g^−1^ dm) and coumarins (6.77 ± 0.04 mg 100 g^−1^ dm). Moreover, seaside habitats generally showed the lowest presence of all polyphenols. Still, based on total polyphenols content, a difference between seaside samples and ones from other two regions can be observed, where continental and mountain samples showed significantly higher levels of total polyphenols when compared to the samples from seaside zone. This could be explained as a plant’s self-defense against oxidative stress caused by lower temperatures. According to Di Virgillo et al. (2015) [1] habitat greatly affects the accumulation of polyphenolic compounds in nettle. Just as in the current study, other authors also confirmed a diversity in nettle polyphenols content in growing areas [7,14].

### 3.2. Influence of Phenological Stage and Habitat on Pigments in Nettle Leaves and Stalks

**Figure 1:**
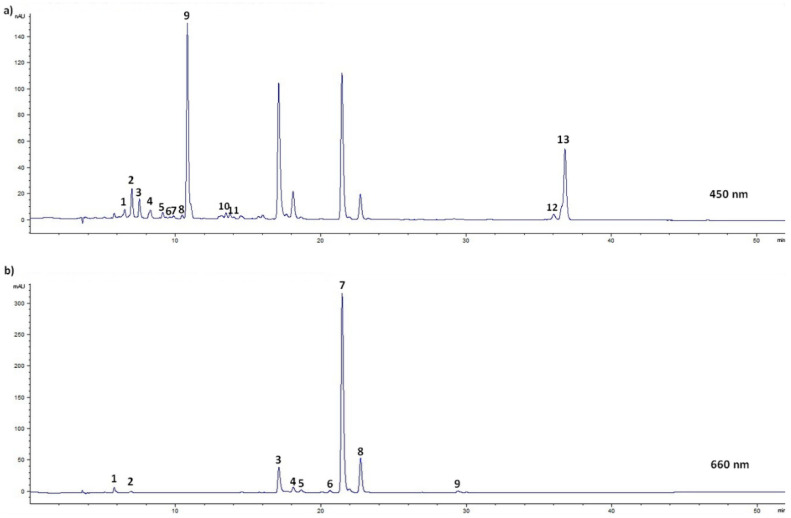
HPLC UV-VIS/PDA detection of pigments in wild nettle leaves (*Urtica dioica* L.) collected from Poreč before flowering: (**a**) at 450 nm (1 = violaxanthin derivative 1, 2 = neoxanthin derivative 1, 3 = neoxanthin, 4 = violaxanthin, 5 = violaxanthin derivative 2, 6 = 13′-*cis*-lutein, 7 = neoxanthin derivative 2, 8 = lutein 5,6-epoxide, 9 = lutein, 10 = zeaxanthin, 11 = 9′-*cis*-lutein, 12 = α-carotene, 13 = β-carotene); (**b**) at 660 nm (1 = chlorophyll *a* derivative 1, 2 = chlorophyll *a* derivative 2, 3 = chlorophyll *b*, 4 = chlorophyll *b* derivative 1, 5 = chlorophyll *a* derivative 3, 6 = chlorophyll *a* derivative 4, 7 = chlorophyll *a*, 8 = chlorophyll *a* derivative 5, 9 = chlorophyll *a* derivative 6).

The presence of nettle natural color carriers, carotenoids and chlorophylls was monitored by HPLC analysis, which has detected a total of 13 carotenoids and 9 chlorophylls in wild nettle leaves and stalks, namely neoxanthin and its two derivatives, violaxanthin and its two derivatives, 13′-*cis*-lutein, lutein 5,6-epoxide, lutein, zeaxanthin, 9′-*cis*-lutein, α-carotene, β-carotene, chlorophyll *a* and its six derivatives and chlorophyll *b* and its derivative (Figure 1, Supplementary file 1). A similar chlorophylls and carotenoids composition was previously reported [4,11]. For statistical purposes, identified pigments were grouped and analyzed as total carotenoids and total chlorophylls, as well as their sum (total pigments) (Table 4). Total pigments GM was 644.22 mg 100 g^−1^ dm, most of which were chlorophylls (GM 611.19 mg 100 g^−1^ dm), while carotenoids were less present (GM 33.03 mg 100 g^−1^ dm). Other authors also reported higher chlorophylls content in nettle leaves extracts in comparison with the content of carotenoids [9,11,47,49].

As presented in Table 4, all sources of variation significantly (*p* < 0.01) affected both groups of pigments as well as their sum. When comparing the pigments distribution in examined plant parts, abundance in pigments was expectedly higher in leaves since they are major photosynthesis organs [50]. Accordingly, Hojnik et al. (2007) [18] also reported a much higher concentration of chlorophylls in nettle leaves in comparison with stalks (147.1 vs. 16 mg g^−1^ extract). Furthermore, determined values for total chlorophylls in leaves were similar to previously reported results by Biesiada et al. (2010) [46], Zeipiņa et al. (2014) [49] and Repajić et al. (2020) [11], but were higher than in Đurović et al.’s (2017) [9] study. Also, the obtained total carotenoids content was in accordance with the values documented in Repajić et al.’s (2020) [11] study, but it showed dissimilarity in comparison with the data of other authors [4,9,46,49], probably due to environmental differences. 

Regarding the phenological stage, the highest amounts of all analyzed pigments were observed during the 2nd stage (flowering), where chlorophylls were the dominant pigments present in almost a 19-fold higher concentration (691.46 mg 100 g^−1^ dm) when compared to the amount of carotenoids (36.97 mg 100 g^−1^ dm). Similarly, Biesiada et al. (2009) [47] reported increased content of chlorophylls and carotenoids in nettle leaves when harvested in July in comparison with the harvest in May. Additionally, Marchetti et al. (2018) [10] observed that the highest lutein and β-carotene concentrations in nettle leaves occurred during the flowering stage (184 and 6.7 μg g^−1^ dm, respectively). Pajević et al. (1999) [51] also determined the maximum levels of chlorophylls and carotenoids in leaves of five alfalfa (*Medicago sativa* L.) genotypes just before and during the flowering stage. These similar patterns can be explained by enhanced production of secondary metabolites, such as plant pigments, during the flowering stage as a plant mechanism for fulfilling important physiological tasks like attracting pollinators [20].

When observing the differences in nettle pigments among the examined habitats, generally samples grown in seaside regions (particularly in the Limski zaljev and Bale habitats) had the highest pigments content. As this area was generally characterized by higher temperatures and lower accumulated precipitation (Table 1), these results are expected since the level of pigments in nettle is influenced by environmental factors, primarily the climate and growing location, where exposure to higher temperatures and more solar energy will result in a higher pigments content [24]. The results of Candido et al.’s (2015) [52] study, in which they examined carotenoid content in buriti palms pulp grown in two different regions (Amazon and Cerrado, Brazil), supported the aforementioned results. They concluded that a higher content of carotenoids was measured in samples from the Amazon area, characterized by higher temperatures and humidity which prevent photodegradation of fruit pigments.

### 3.3. Influence of Phenological Stage and Habitat on Antioxidant Capacity in Nettle Leaves and Stalks

The results of nettle antioxidant capacity measured by the ORAC method are given in Table 4 and Supplementary file 1. ORAC GM was 9.67 mmol TE 100 g^−1^ dm. Moreover, the nettle antioxidant capacity was significantly influenced (*p* < 0.01) by all examined sources of variation. Nettle leaves showed higher antioxidant capacity in comparison with stalks (11.96 mmol TE 100 g^−1^ dm vs. 7.37 mmol TE 100 g^−1^ dm). Similar ORAC values in nettle leaves were recorded in study of Repajić et al. (2020) [11], while Česlova et al. (2016) [53] obtained the same results by measuring the antioxidant capacity of different nettle parts infusions, where nettle leaves gained higher DPPH levels when compared to stalks. In support, Kırca and Arslan (2008) [54] concluded that leaves and flowers of different examined plants had a higher antioxidant capacity when compared to stalks and seeds.

When observing the influence of phenological stage, the highest ORAC value was observed during flowering, after which it significantly decreased and was the lowest after flowering. Similar to the results of the current study, other authors [19,46] documented that the antioxidant capacity of nettle leaves was higher in the earliest periods (April/May and June/July), after which it decreased (September/October).

Nettle samples showed diversity in antioxidant capacity upon habitat variations. As can be observed, samples from the continental and mountain part were described with the highest ORAC levels as opposed to nettles grown in seaside areas, which were characterized with the lowest antioxidant capacity levels. These results are in accordance with previously discussed contents of polyphenols and pigments, where a certain grouping of the samples according to the presence of polyphenols and pigments by the growing area is evident. Moreover, calculated correlation coefficients supported this observation, since they showed a strong correlation between ORAC values and cinnamic acids, flavonols and total phenols (Table 5).

Obtained results clearly demonstrated the importance of the appropriate plant part selection as well as its phenological stage with the presence of the highest bioactive compounds accumulation in order to obtain the maximally enriched product, which will be beneficial for consumers.

### 3.4. PCA Analysis

**Figure 2:**
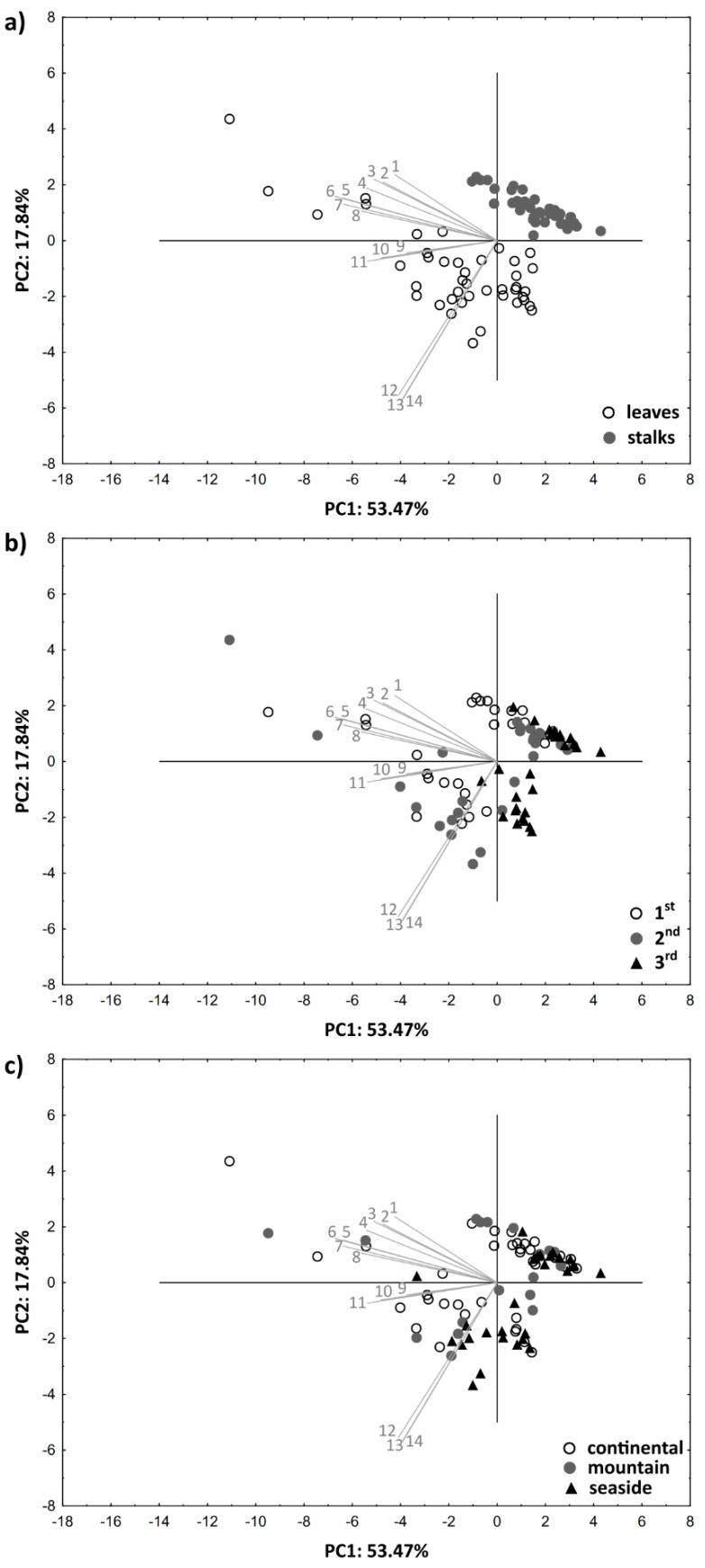
Distribution of wild nettle samples in two-dimensional coordinate system defined by the first two principal components (PC1 and PC2) according to the (**a**) plant part; (**b**) phenological stage; (**c**) growing region (1 = isoflavones, 2 = flavanones, 3 = flavones, 4 = benzoic acids, 5 = cinnamic acids, 6 = total polyphenols, 7 = flavonols, 8 = flavan-3-ols, 9 = other acids, 10 = coumarins, 11 = ORAC, 12 = carotenoids, 13 = chlorophylls, 14 = total pigments).

Additionally, in order to examine a possible grouping of the nettle samples according to the applied sources of variations, PCA was carried out and obtained results are presented in Figure 2.

According to the preliminary PCA, a communality value of ≥0.5 described all 14 variables, thus they were all included in the test. The first two components (PC1 and PC2) explained 71.31% of total variance, where PC1 accounted for 53.47% of total variance, while PC2 attributed to 17.84% of total variance. Since PC1 strongly/very strongly negatively correlated (−0.77 ≤ r ≤ −0.96) with benzoic and cinnamic acids, flavonols, flavan-3-ols, flavones, ORAC values and total polyphenols, while PC2 had a strong/very strong correlation with carotenoids, chlorophylls and total pigments (−0.79 ≤ r ≤ −0.81), these variables could be considered as the most discriminating variables.

As can be seen in Figure 2a, separation of the samples clearly occurs based on the plant part. Most of the leaf samples were distributed at negative PC2 values, while all samples of stalks were situated at positive PC2 values. Regarding the phenological stage, a certain grouping appeared between samples from the 1st and 3rd phenological stage, where samples collected before flowering were mainly situated at negative PC1 values and almost all of the post-flowering samples were located at the positive PC1 values (Figure 2b). a partial grouping of nettle samples is visible in Figure 2c based on the growing region, where the most of separation can be seen to be present between continental and seaside samples, although this did not completely occur.

## 4. Conclusions

The current study confirmed the abundance of wild nettle with diverse bioactive molecules such as low molecular weight polyphenols and pigments, where 41 phenolic compounds, 13 carotenoids and 9 chlorophylls were documented. By using applied extraction conditions, cinnamic acids and flavonols were found to be the dominant classes of identified polyphenols (33.10–519.81 mg 100 g^−1^ dm and 57.44–383.25 mg 100 g^−1^ dm, respectively), while chlorophylls were the most abundant natural pigments (4.26–1934.38 mg 100 g^−1^ dm). Moreover, the ORAC values of obtained nettle extracts ranged from 3.05 to 19.83 mmol TE 100 g^−1^ dm. However, in order to obtain high valuable wild nettle extracts that are abundant in natural antioxidants, it is of the utmost importance to select appropriate plant parts as well as an appropriate harvest time. Obtained results evidenced that the highest levels of nettle bioactives accompanied by high antioxidant capacity were present in leaves, which should be collected during the early phenological period (before and at the flowering stage). Moreover, the amounts of wild nettle polyphenols and pigments greatly differed based on the natural habitat, as samples from the seaside region were characterized with elevated accumulation of pigments, while higher polyphenols amounts were present in habitats located in continental and mountain areas. This research will surely contribute to the selection of plant part and phenological stage for nettle optimal harvest, as well as to designate nettle natural habitats that have been shown to be a source of valuable plant material. These findings present the basis for the production of nettle seedlings with high bioactives content, which could further be used in the production of liquid and dry extracts. Furthermore, they showed the importance of a multidisciplinary approach for the selection of a plant part as well as its phenological stage in order to provide highly enriched products intended for the benefit of consumers.

In addition, besides low molecular weight polyphenols and pigments covered by this research, future studies could also include other beneficial compounds present in nettle such as oligomers and polymers as well as sterols, to provide a full insight into the nettle’s bioactive potential.

## Figures and Tables

**Table 1:** Location and weather characteristics of wild nettle (*Urtica dioica* L.) habitats.

Region	Location	Altitude/ Latitude/Longitude	Weather Parameters	Phenological Stage					
				I Before Flowering		II During Flowering		III After Flowering	
				April	May	June	July	September	October
Continental	Sela Žakanjska	244 m 45°36′27.80′′N 15°20′38.21′′E	a.d. T (°C)	11.0	13.4	22.6	22.0	16.3	12.7
			T min (°C)	−0.1	0.4	11.4	9.2	2.9	0.7
			T max (°C)	27.7	26.5	34.7	35.6	30.5	26.7
			a.p. (mm)	143.4	170.1	73.8	85.4	101.8	55.6
	Sopčić Vrh	177 m 45°34′14.88′′N 15°20′24.98′′E	a.d. T (°C)	11.0	13.4	22.6	22.0	16.3	12.7
			T min (°C)	−0.1	0.4	11.4	9.2	2.9	0.7
			T max (°C)	27.7	26.5	34.7	35.6	30.5	26.7
			a.p. (mm)	143.4	170.1	73.8	85.4	101.8	55.6
	Žakanje	178 m 45°36′34.38′′N 15°20′14.96′′E	a.d. T (°C)	11.0	13.4	22.6	22.0	16.3	12.7
			T min (°C)	−0.1	0.4	11.4	9.2	2.9	0.7
			T max (°C)	27.7	26.5	34.7	35.6	30.5	26.7
			a.p. (mm)	143.4	170.1	73.8	85.4	101.8	55.6
	Zagreb I (Gračani)	119 m 45°51′31.10′′N 15°58′19.34′′E	a.d. T (°C)	12.4	13.7	23.8	22.9	17.2	13.2
			T min (°C)	1.9	2.1	13.3	10.4	4.6	2.2
			T max (°C)	27.1	26.1	34.6	35.9	33.1	25.9
			a.p. (mm)	81.1	147.7	70.8	76.8	150.1	42.3
	Zagreb II (Vrapče)	119 m 45°49′8.69′′N 15°52′49.84′′E	a.d. T (°C)	13.6	14.3	24.8	24.1	18.4	14.8
			T min (°C)	5.4	5.6	15.1	14.0	8.5	5.2
			T max (°C)	22.9	27.1	27.3	35.5	32.7	24.6
			a.p. (mm)	85.2	123.1	83.9	65.8	131.6	39.5
	Koretići	410 m 45°48′47.23′′N 15°33′36.18′′E	a.d. T (°C)	9.0	9.9	20.4	19.6	14.5	13.3
			T min (°C)	−3.0	−1.8	9.8	7.2	1.5	3.5
			T max (°C)	22.4	21.5	31.2	30.0	28.2	22.8
			a.p. (mm)	135.7	283.7	81.4	184.8	120.2	59.5
Mountain	Ogulin	320 m 45°15′47.84′′N 15°13′42.36′′E	a.d. T (°C)	10.8	12.4	21.6	21.4	15.6	13.0
			T min (°C)	0.5	0.5	11.8	8.3	2.8	3.0
			T max (°C)	25.5	25.1	33.4	33.0	29.3	25.9
			a.p. (mm)	167.4	319.2	139.5	109.4	143.6	64.2
	Čovići I	456 m 44°49′44.07′′N 15°17′57.29′′E	a.d. T (°C)	9.4	11.1	20.1	19.7	14.0	10.6
			T min (°C)	−2.1	−1.3	7.6	5.5	−2.0	1.2
			T max (°C)	24.8	25.0	34.1	34.5	29.6	25.1
			a.p. (mm)	138.6	189.3	25.1	106.2	106.9	31.8
	Čovići II	456 m 44°49′50.05′′N 15°17′57.18′′E	a.d. T (°C)	9.4	11.1	20.1	19.7	14.0	10.6
			T min (°C)	−2.1	−1.3	7.6	5.5	−2.0	1.2
			T max (°C)	24.8	25.0	34.1	34.5	29.6	25.1
			a.p. (mm)	138.6	189.3	25.1	106.2	106.9	31.8
Seaside	Poreč	0.34 m 45°13′37.03′′N 13°35′39.64′′E	a.d. T (°C)	13.0	14.5	24.3	24.9	19.4	15.7
			T min (°C)	3.9	6.0	13.2	13.4	7.3	6.3
			T max (°C)	23.5	22.7	33.6	33.6	30.9	25.7
			a.p. (mm)	116.1	210.0	7.3	58.7	143.2	38.6
	Limski zaljev	17 m 45°7′56.45′′N 13°39′13.78′′E	a.d. T (°C)	13.0	14.5	24.3	24.9	19.4	15.7
			T min (°C)	3.9	6.0	13.2	13.4	7.3	6.3
			T max (°C)	23.5	22.7	33.6	33.6	30.9	25.7
			a.p. (mm)	116.1	210.0	7.3	58.7	143.2	38.6
	Bale	129 m 45°2′25.93′′N 13°47′8.88′′E	a.d. T (°C)	13.4	14.4	23.9	24.5	19.8	15.5
			T min (°C)	4.9	4.5	13.8	13.6	7.5	5.8
			T max (°C)	23.7	24.5	34.0	34.3	33.0	25.5
			a.p. (mm)	129.5	264.7	37.4	71.5	91.1	42.0
	Vodnjan	141 m 44°57′28.79′′N 13°51′6.10′′E	a.d. T (°C)	13.4	14.4	23.9	24.5	19.8	15.5
			T min (°C)	4.9	4.5	13.8	13.6	7.5	5.8
			T max (°C)	23.7	24.5	34.0	34.3	33.0	25.5
			a.p. (mm)	129.5	264.7	37.4	71.5	91.1	42.0
	Muntrilj	342 m 45°14′30.84′′N 13°48′38.44′′E	a.d. T (°C)	11.1	12.5	22.2	22.3	16.4	13.1
			T min (°C)	0.5	1.1	11.2	9.5	2.3	2.5
			T max (°C)	23.3	23.5	35.8	36.1	31.7	25.2
			a.p. (mm)	135.1	295.1	26.0	72.6	90.5	26.4

a.d. T = average day temperature, T min = minimal day temperature, T max = maximal day temperature, a.p. = accumulated precipitation.

**Table 2:** Mass spectrometric data and identification of polyphenols.

Compound	Rt (min)	Cone Voltage (V)	Collision Energy (V)	Ionization Mode	Precursor Ion (*m/z*)	Fragment Ions (*m/z*)	Tentative Identification
Benzoic acids							
**2**	0.828	105	9	-	153	109	Protocatechuic acid (3,4-dihydoxybenzoic acid)
**14**	0.992	100	9	-	153	109	Gentisic acid (2,5-dihydroxybenzoic acid)
**31**	8.837	90	7	-	197	182	Syringic acid
**34**	11.358	80	10	-	137	93	*p*-hydroxybenzoic acid
**35**	11.375	100	10	-	169	125	Gallic acid *
Cinnamic acids							
**12**	0.975	80	10	-	179	135	Caffeic acid *
**15**	1.254	80	10	-	353	191	Chlorogenic acid *
**19**	3.332	80	10	-	163	119	*p*-coumaric acid *
**21**	4.490	100	5	-	147	103	Cinnamic acid
**25**	6.158	80	5	-	193	178	Ferulic acid *
**32**	11.012	100	17	-	223	193	Sinapic acid *
Other phenolic acids							
**16**	1.620	150	20	-	191	85	Quinic acid *
Flavonols							
**3**	0.842	120	15	+	625	317	Isorhamnetin rutinoside
**4**	0.856	120	15	+	595	287	Kaempferol-3-rutinoside *
**5**	0.880	100	5	+	449	303	Quercetin rhamnoside
**6**	0.880	30	5	+	449	287	Kaempferol hexoside
**8**	0.907	140	25	+	319	273	Myricetin *
**10**	0.938	130	15	-	301	151	Quercetin
**17**	1.855	100	5	+	465	303	Quercetin-3-glucoside *
**18**	2.461	120	5	+	611	303	Quercetin-3-rutinoside *
**24**	5.963	160	21	-	315	300	Isorhamnetin
**27**	7.106	100	5	+	419	287	Kaempferol pentoside
**28**	7.256	100	5	+	435	303	Quercetin pentoside
**29**	7.930	100	5	+	433	287	Kaempferol rhamnoside
**30**	8.242	100	10	+	507	303	Quercetin acetylhexoside
**33**	11.232	100	15	+	653	303	Quercetin acetylrutinoside
**36**	11.391	100	15	+	597	303	Quercetin pentosylhexoside
**39**	11.758	120	15	+	581	287	Kaempferol pentosylhexoside
**41**	11.822	130	0	-	285	285	Kaempferol
Flavan-3-ols							
**23**	4.728	100	5, 15	+	459	289, 139	Epigallocatechin gallate *
**37**	11.615	100	10	+	291	139	Epicatechin *
**38**	11.621	100	5	+	291	165	Catechin *
**40**	11.792	100	5	+	443	291	Epicatechin gallate *
Flavones							
**7**	0.890	135	5	+	433	271	Apigenin hexoside
**9**	0.924	140	35	+	287	153	Luteolin *
**22**	4.615	80	30	+	271	153	Apigenin *
Isoflavones							
**20**	4.468	145	32	-	269	133	Genistein
Flavanones							
**11**	0.945	130	16	-	271	151	Naringenin *
Coumarins							
**1**	0.821	120	19	-	161	133	Umbelliferone (7-hydroxycoumarin)
**13**	0.979	105	15	-	177	133	Esculetin *
**26**	6.333	80	8	-	191	176	Scopoletin *

* Identification confirmed using authentic standards.

**Table 3:** The differences in polyphenols content (mg 100 g^−1^ dm) in wild nettle (*Urtica dioica* L.) due to the plant part, phenological stage and habitat.

Source of Variation		Benzoic Acids	Cinnamic Acids	Other Acids	Flavonols	Flavan-3-ols	Flavones	Isoflavones	Flavanones	Coumarins	Total Polyphenols
**Plant part**		*p* < 0.01 *	*p* < 0.01 *	*p* < 0.01 *	*p* < 0.01 *	*p* < 0.01 *	*p* < 0.01 *	*p* < 0.01 *	*p* < 0.01 *	*p* < 0.01 *	*p* < 0.01 *
leaves		12.55 ± 0.04b	209.46 ± 0.26b	4.30 ± 0.03b	160.26 ± 0.14b	25.99 ± 0.04b	29.28 ± 0.05b	3.37 ± 0.02b	0.40 ± 0.01b	6.53 ± 0.01b	452.14 ± 0.39b
stalks		7.86 ± 0.04a	148.98 ± 0.26a	1.45 ± 0.03a	108.94 ± 0.14a	15.42 ± 0.04a	19.84 ± 0.05a	2.81 ± 0.02a	0.29 ± 0.01a	4.09 ± 0.01a	309.67 ± 0.39a
**Phenological stage**		*p* < 0.01 *	*p* < 0.01 *	*p* < 0.01 *	*p* < 0.01 *	*p* < 0.01 *	*p* < 0.01 *	*p* < 0.01 *	*p* < 0.01 *	*p* < 0.01 *	*p* < 0.01 *
1st		12.65 ± 0.05c	223.32 ± 0.32c	3.66 ± 0.03c	169.53 ± 0.17c	22.23 ± 0.05b	31.89 ± 0.06c	3.70 ± 0.02c	0.48 ± 0.01c	7.28 ± 0.02c	474.75 ± 0.48c
2nd		11.55 ± 0.05b	202.70 ± 0.32b	3.18 ± 0.03b	141.72 ± 0.17b	23.88 ± 0.05c	22.28 ± 0.06b	2.99 ± 0.02b	0.33 ± 0.01b	5.11 ± 0.02b	413.75 ± 0.48b
3rd		6.42 ± 0.05a	111.63 ± 0.32a	1.78 ± 0.03a	92.54 ± 0.17a	16.00 ± 0.05a	19.50 ± 0.06a	2.58 ± 0.02a	0.22 ± 0.01a	3.53 ± 0.02a	254.21 ± 0.48a
**Region/Habitat**		*p* < 0.01 *	*p* < 0.01 *	*p* < 0.01 *	*p* < 0.01 *	*p* < 0.01 *	*p* < 0.01 *	*p* < 0.01 *	*p* < 0.01 *	*p* < 0.01 *	*p* < 0.01 *
C	Sela Žakanjska	12.06 ± 0.10g	200.25 ± 0.70h	2.10 ± 0.07b	134.87 ± 0.38g	16.68 ± 0.11b	24.18 ± 0.12g	2.92 ± 0.05d	0.31 ± 0.02bcd	4.41 ± 0.04a	397.78 ± 1.03g
	Sopčić Vrh	9.11 ± 0.10cd	215.63 ± 0.70i	4.12 ± 0.07f	150.83 ± 0.38i	26.97 ± 0.11i	26.69 ± 0.12h	3.25 ± 0.05e	0.49 ± 0.02fg	5.20 ± 0.04d	442.29 ± 1.03j
	Žakanje	19.39 ± 0.10i	227.10 ± 0.70j	2.56 ± 0.07c	177.87 ± 0.38j	32.13 ± 0.11j	42.44 ± 0.12j	5.29 ± 0.05i	0.41 ± 0.02def	5.92 ± 0.04f	513.12 ± 1.03l
	Zagreb I	10.21 ± 0.10e	172.62 ± 0.70e	4.22 ± 0.07fg	130.21 ± 0.38f	15.63 ± 0.11a	23.33 ± 0.12f	1.95 ± 0.05a	0.33 ± 0.02cde	6.76 ± 0.04h	365.26 ± 1.03e
	Zagreb II	11.06 ± 0.10f	185.09 ± 0.70f	4.21 ± 0.07fg	125.81 ± 0.38d	18.80 ± 0.11cd	21.49 ± 0.12d	3.47 ± 0.05ef	0.31 ± 0.02bcd	6.51 ± 0.04g	376.74 ± 1.03f
	Koretići	10.87 ± 0.10f	195.03 ± 0.70g	4.53 ± 0.07gh	144.36 ± 0.38h	22.09 ± 0.11g	18.87 ± 0.12b	3.77 ± 0.05g	0.45 ± 0.02efg	5.06 ± 0.04d	405.02 ± 1.03h
M	Ogulin	13.18 ± 0.10h	212.80 ± 0.70i	4.62 ± 0.07h	182.65 ± 0.38k	20.88 ± 0.11f	36.09 ± 0.12i	3.51 ± 0.05f	0.56 ± 0.02g	6.77 ± 0.04h	481.06 ± 1.03k
	Čovići I	9.44 ± 0.10cd	203.51 ± 0.70h	3.73 ± 0.07e	152.62 ± 0.38i	19.69 ± 0.11e	23.92 ± 0.12fg	4.47 ± 0.05h	0.44 ± 0.02efg	5.63 ± 0.04e	423.46 ± 1.03i
	Čovići II	8.96 ± 0.10c	194.18 ± 0.70g	3.08 ± 0.07d	127.91 ± 0.38e	21.62 ± 0.11g	17.14 ± 0.12a	2.91 ± 0.05d	0.53 ± 0.02g	4.70 ± 0.04bc	381.01 ± 1.03f
S	Poreč	9.37 ± 0.10cd	130.43 ± 0.70a	1.31 ± 0.07a	107.51 ± 0.38a	16.43 ± 0.11b	20.64 ± 0.12c	1.87 ± 0.05a	0.16 ± 0.02a	4.40 ± 0.04a	292.12 ± 1.03a
	Limski zaljev	9.53 ± 0.10d	141.67 ± 0.70c	1.07 ± 0.07a	111.21 ± 0.38b	18.32 ± 0.11c	23.43 ± 0.12f	2.36 ± 0.05b	0.23 ± 0.02abc	4.80 ± 0.04c	312.61 ± 1.03c
	Bale	7.14 ± 0.10b	143.55 ± 0.70c	1.82 ± 0.07b	114.39 ± 0.38c	19.17 ± 0.11de	22.41 ± 0.12e	2.68 ± 0.05cd	0.16 ± 0.02a	4.49 ± 0.04a	315.81 ± 1.03cd
	Vodnjan	6.14 ± 0.10a	134.44 ± 0.70b	1.03 ± 0.07a	112.89 ± 0.38bc	22.74 ± 0.11h	22.13 ± 0.12e	2.35 ± 0.05b	0.19 ± 0.02ab	5.15 ± 0.04d	307.05 ± 1.03b
	Muntrilj	6.40 ± 0.10a	152.75 ± 0.70d	1.87 ± 0.07b	111.27 ± 0.38b	18.72 ± 0.11cd	21.06 ± 0.12cd	2.46 ± 0.05bc	0.22 ± 0.02abc	4.55 ± 0.04ab	319.29 ± 1.03d
**Grand mean**		**10.20**	**179.22**	**2.88**	**134.60**	**20.70**	**24.56**	**3.09**	**0.34**	**5.31**	**380.90**

C = continental, M = mountain, S = seaside. * Statistically significant variable at *p* ≤ 0.05. Results are expressed as mean ± SE (N = 4). Values with different letters within column are statistically different at *p* ≤ 0.05.

**Table 4:** The differences in pigments content (mg 100 g^−1^ dm) and ORAC values (mmol TE 100 g^−1^ dm) in wild nettle (*Urtica dioica* L.) upon plant part, phenological stage and habitat.

Source of Variation		Carotenoids	Chlorophylls	Total Pigments	ORAC
**Plant Part**		*p* < 0.01 *	*p* < 0.01 *	*p* < 0.01 *	*p* < 0.01 *
leaves		61.46 ± 0.08b	1126.94 ± 0.66b	1188.40 ± 0.71b	11.96 ± 0.02b
stalks		4.60 ± 0.08a	95.45 ± 0.66a	100.05 ± 0.71a	7.37 ± 0.02a
**Phenological Stage**		*p* < 0.01 *	*p* < 0.01 *	*p* < 0.01 *	*p* < 0.01 *
1st		32.49 ± 0.10b	589.07 ± 0.81b	621.56 ± 0.86b	11.26 ± 0.04b
2nd		36.97 ± 0.10c	691.46 ± 0.81c	728.44 ± 0.86c	12.10 ± 0.04c
3rd		29.64 ± 0.10a	553.04 ± 0.81a	582.67 ± 0.86a	5.63 ± 0.04a
**Region/Habitat**		*p* < 0.01 *	*p* < 0.01 *	*p* < 0.01 *	*p* < 0.01 *
C	Sela Žakanjska	37.29 ± 0.21h	701.89 ± 1.76g	739.18 ± 1.87h	11.76 ± 0.06i
	Sopčić Vrh	31.95 ± 0.21d	558.11 ± 1.76c	590.06 ± 1.87d	11.78 ± 0.06j
	Žakanje	27.94 ± 0.21c	466.84 ± 1.76a	494.78 ± 1.87a	12.25 ± 0.06m
	Zagreb I	26.70 ± 0.21b	480.27 ± 1.76b	506.97 ± 1.87b	11.89 ± 0.06k
	Zagreb II	32.14 ± 0.21d	600.73 ± 1.76d	632.87 ± 1.87e	9.46 ± 0.06f
	Koretići	31.81 ± 0.21d	596.83 ± 1.76d	628.64 ± 1.87e	11.22 ± 0.06h
M	Ogulin	33.23 ± 0.21e	598.67 ± 1.76d	631.91 ± 1.87e	12.20 ± 0.06l
	Čovići I	25.65 ± 0.21a	472.09 ± 1.76ab	497.74 ± 1.87a	10.59 ± 0.06g
	Čovići II	34.29 ± 0.21f	650.39 ± 1.76e	684.68 ± 1.87f	9.46 ± 0.06f
S	Poreč	27.40 ± 0.21bc	552.88 ± 1.76c	580.28 ± 1.87c	6.28 ± 0.06b
	Limski zaljev	40.21 ± 0.21j	719.69 ± 1.76h	759.90 ± 1.87i	6.26 ± 0.06a
	Bale	38.35 ± 0.21i	760.95 ± 1.76i	799.30 ± 1.87j	6.58 ± 0.06c
	Vodnjan	35.93 ± 0.21g	678.67 ± 1.76f	714.60 ± 1.87g	8.08 ± 0.06e
	Muntrilj	39.55 ± 0.21j	718.67 ± 1.76h	758.23 ± 1.87i	7.52 ± 0.06d
**Grand mean**		**33.03**	**611.19**	**644.22**	**9.67**

C = continental, M = mountain, S = seaside. * Statistically significant variable at *p* ≤ 0.05. Results are expressed as mean ± SE (N = 4). Values with different letters within column are statistically different at *p* ≤ 0.05.

**Table 5:** Pearson’s correlations between analyzed compounds (mg 100 g^−1^ dm) and ORAC values (mmol TE 100 g^−1^ dm).

Group of Compounds	ORAC Value
Benzoic acids	0.53 *
Cinnamic acids	0.71 *
Other acids	0.59 *
Flavonols	0.68 *
Flavan-3-ols	0.47 *
Flavones	0.36 *
Isoflavones	0.36 *
Flavanones	0.39 *
Coumarins	0.60 *
Total phenols	0.71 *
Carotenoids	0.46 *
Chlorophylls	0.44 *
Total pigments	0.44 *

* *p* ≤ 0.05.

## Supplementary Materials

The following figures and tables are available online at https://www.mdpi.com/2304-8158/10/1/190/s1, (file 1) Figure S1: LC-MS/MS chromatogram in dMRM acquisition from the extract of wild nettle leaves (*Urtica dioica* L.) collected from Poreč before flowering, (file 2) Tables S1–S3: Concentrations of individual compounds and ORAC values in nettle (*Urtica dioica* L.) samples.
