# Supplementary material for: Bioactive Compounds in Wild Nettle (Urtica dioica L.) Leaves and Stalks: Polyphenols and Pigments upon Seasonal and Habitat Variations
Source: Foods. 2021 Jan 18;10(1):190. doi: 10.3390/foods10010190 (PMC7831946; doi:10.3390/foods10010190)
Supplement: Supplementary file 1 [file foods-10-00190-s001.zip › Supplementary file 2.pdf]

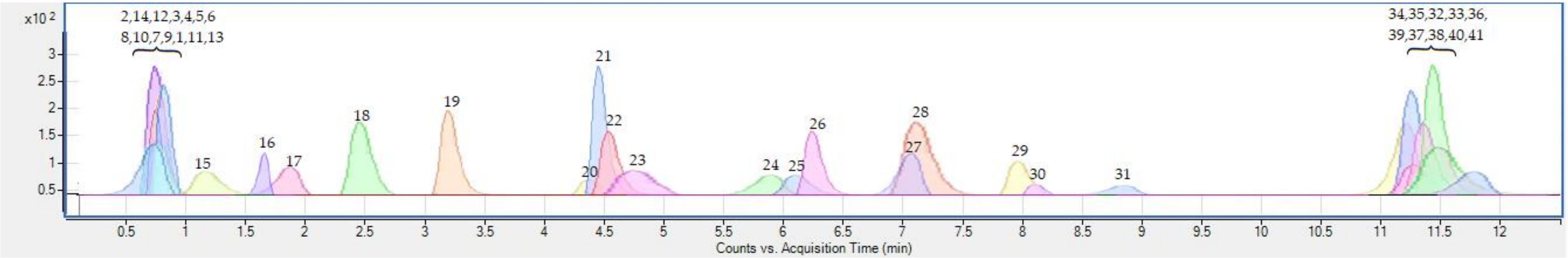

Supplementary Figure 1. LC-MS/MS chromatogram in dMRM acqusition from extract of wild nettle leaves (*Urtica dioica* L.) collected from Poreč before flowering. Peak designation was: (1) Umbelliferone, (2) Protocatechuic acid, (3) Isorhamnetin rutinoside, (4) Kaempferol-3-rutinoside, (5) Quercetin rhamnoside, (6) Kaempferol hexoside, (7) Apigenin hexoside, (8) Myricetin, (9) Luteolin, (10) Quercetin, (11) Naringenin, (12) Caffeic acid, (13) Esculetin, (14) Gentisic acid, (15) Chlorogenic acid, (16) Quinic acid, (17) Quercetin-3-glucoside, (18) Quercetin-3-rutinoside, (19) *p*-coumaric acid, (20) Genistein, (21) Cinnamic acid, (22) Apigenin, (23) Epigallocatechin gallate, (24) Isorhamnetin, (25) Ferulic acid, (26) Scopoletin, (27) Kaempferol pentoside, (28) Quercetin pentoside, (29) Kaempferol rhamnoside, (30) Quercetin acetylhexoside (31) Syringic acid, (32) Sinapic acid, (33) Quercetin acetylrutinoside (34) *p*-hydroxybenzoic acid, (35) Gallic acid, (36) Quercetin pentosylhexoside, (37) Epicatechin, (38) Catechin, (39) Kaempferol pentosylhexoside, (40) Epicatechin gallate, (41) Kaempferol
